# *Aegle marmelos* Mediated Green Synthesis of Different Nanostructured Metal Hexacyanoferrates: Activity against Photodegradation of Harmful Organic Dyes

**DOI:** 10.1155/2016/2715026

**Published:** 2016-02-29

**Authors:** Vidhisha Jassal, Uma Shanker, B. S. Kaith

**Affiliations:** Department of Chemistry, Dr. B. R. Ambedkar National Institute of Technology, Jalandhar, Punjab 144011, India

## Abstract

Prussian blue analogue potassium metal hexacyanoferrate (KMHCF) nanoparticles Fe_4_[Fe(CN)_6_]_3_ (FeHCF), K_2_Cu_3_[Fe(CN)_6_]_2_ (KCuHCF), K_2_Ni[Fe(CN)_6_]·3H_2_O (KNiHCF), and K_2_Co[Fe(CN)_6_] (KCoHCF) have been synthesized using plant based biosurfactant* Aegle marmelos* (Bael) and water as a green solvent. It must be emphasized here that no harmful reagent or solvent was used throughout the study. Plant extracts are easily biodegradable and therefore do not cause any harm to the environment. Hence, the proposed method of synthesis of various KMHCF nanoparticles followed a green path. The synthesized nanoparticles were characterized by powder X-ray diffraction (PXRD), Field-Emission Scanning Electron Microscopy (FE-SEM), Transmission Electron Microscopy (TEM), and Fourier Transform Infrared Spectroscopy (FT-IR). MHCF nanoparticles were used for the photocatalytic degradation of toxic dyes like Malachite Green (MG), Eriochrome Black T (EBT), Methyl Orange (MO), and Methylene Blue (MB). Under optimized reaction conditions, maximum photocatalytic degradation was achieved in case of KCuHCF nanoparticles mediated degradation process (MG: 96.06%, EBT: 83.03%, MB: 94.72%, and MO: 63.71%) followed by KNiHCF (MG: 95%, EBT: 80.32%, MB: 91.35%, and MO: 59.42%), KCoHCF (MG: 91.45%, EBT: 78.84%, MB: 89.28%, and MO: 58.20%).

## 1. Introduction

In last few years, Prussian Blue (PB) and its analogues have gained worldwide attraction because of the presence of unique properties like electrochromic, electrochemical, and magnetic properties [1]. Metal hexacyanoferrates are generally denoted with the general formula M_*k*_[Fe(CN)_6_]·*x*H_2_O where M is a transition metal in the high spin and Fe in the low spin configuration. Properties of such complexes have been found to change with the replacement of outer coordination sphere transition metal with another. Metal-to-metal charge transfer between the two transition metal ions exhibits the redox behavior. Catala et al. reported that nano range PB analogues show unique characteristics as compared to their bulk form; for example, nickel hexacyanoferrate nanoparticles possessed super-paramagnetic property [2]. Effect of nanosize in magnetic phase transition temperature with PB nanoparticles was found [3, 4] and owing to such outstanding properties these nanostructures can be applied in various fields like electrochemistry [5–7], optics [8] and molecular magnets [9–12], and battery electrode materials [13–15].

PB analogues can be synthesized using various techniques such as sol-gel or using surfactants like cetyltrimethyl ammonium bromide (CTAB), ethylene diaminetetraacetate (EDTA) or polymers such as poly(vinylpyrrolidone), or in presence of mesostructured silica, porous alumina, and nafion [16–18].

Currently, the use of green nanotechnology is growing worldwide as it involves wide variety of pathways that eliminate the use of toxic substances in order to restore the environment. One of the potential promising methods for the synthesis of nanoparticles employs use of multifunctional nontoxic reactants derived from various parts of plants, such as plant extracts [19], tissues [20], exudates [21], and other parts of the living plants [22]. These parts of the plants are easily available and do not harm the tree itself. Moreover, storage of these parts of plants is very easy. Hence, plant based green protocols are hazard-free, environmentally safer, simple, and fast [23].

The use of such biomaterials for the formation of nanoscale inorganic substances is an alternative to the chemical methods [24]. Various scientists worldwide have carried out research in this new emerging field of nanochemistry and they have successfully synthesized nanoparticles using biogenic source. For example, Gardea-Torresdey et al. reported for the first time synthesis of gold nanoparticles inside live alfalfa plant [25]. Similarly, the same group in 2003 demonstrated the use of alfalfa sprouts for the synthesis of silver nanoparticles [26].

Recently, Ahmed et al. applied a simple and rapid approach for the synthesis of silver nanoparticles using* Azadirachta indica* aqueous leaf extract [27]. Biosynthesis of nanoparticles using different plant species like* Aloe vera* [28],* Cinnamomum camphora* [29],* Capsicum annuum* L. [30],* Medicago sativa* and* Brassica juncea* [31],* Brassica chicory* [32],* Azadiracta indica* [33] and* Cymbopogon flexuous* [34],* Ficus Microcarpa* [35],* Clitoria ternatea* and* Solanum nigrum* [36],* Prosopis farcta* [37], and* Dalbergia spinosa* [38] has been successfully carried out in the last few decades.

**Figure 1:**
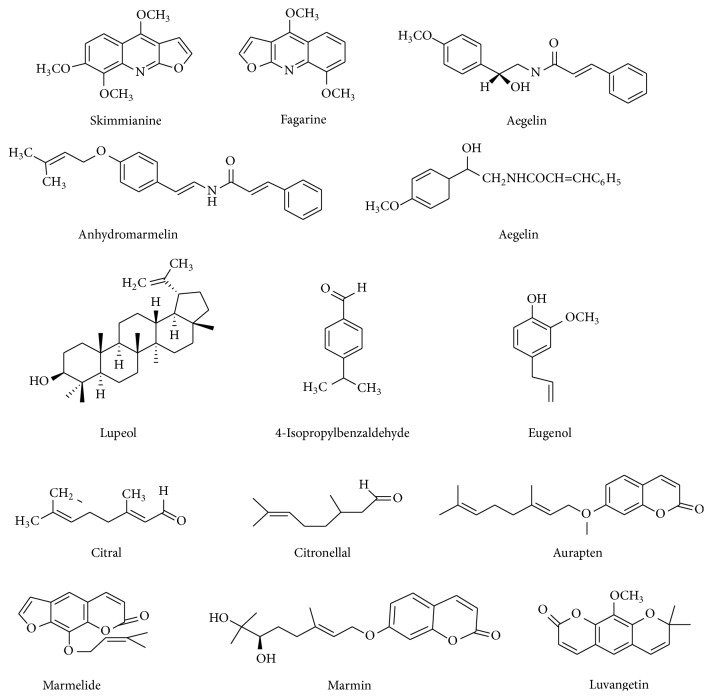
Structures of various bioactive compounds present in* Aegle marmelos* [40].

The present research reports on the use of* Aegle marmelos* (Bael) leaf extract as a natural plant extract for the synthesis of various metal hexacyanoferrate (KMHCF) nanoparticles.* Aegle marmelos* is known from prehistoric times for its diverse importance in several fields such as medicinal, commercial, nutritional, and environmental [39]. The leaves of this tree mainly contain polyphenols such as alkaloids, terpenoids, and phenylpropanoids (Figure 1) [40]. These leaves are found to be well recognized for their healing power against wide variety of bacterial and fungal infections [39, 41]. Recently, Rao and Paria reported one-pot synthesis of silver nanoparticles of average size ~60 nm using* Aegle marmelos* leaf extract [42]. The same group used* Aegle marmelos* leaf extract for the synthesis of Au and Ag nanoparticles by optimising process parameters using Taguchi method [43]. Furthermore, Kumar et al. carried out a facile precipitation route for the green synthesis of zinc oxysulfide quantum dots using* Aegle marmelos *fruit extract [44]. This eco-friendly approach encouraged the authors to use* Aegle marmelos* as a biosurfactant for the synthesis of different KMHCF nanoparticles.

The rapid pace of industrialisation and its toxic releases into the water effluents have strongly impacted the balance of nature. This makes waste water treatment an important issue. In developing countries, industries are a major source for an economic boost. Dyes play an important role in various industries such as printing, paper, textile, printing, and cosmetics. For processing 1000 kg of clothes, approximately 1000 L of water is required. Their discharge into the water bodies without any treatment causes direct threat to the eco-system. In search of a friendly method, we carried out the successful removal of harmful organic dyes from waste water. In this study, plant-derived natural surfactant,* Aegle marmelos*, was used for the synthesis of various KMHCF nanoparticles. Activity of the synthesized KMHCF nanoparticles was evaluated for the photocatalytic degradation of harmful organic dyes namely, Malachite Green (MG), Eriochrome Black T (EBT), Methyl Orange (MO), and Methylene Blue (MB).

## 2. Material and Methods

### 2.1. Chemicals

Potassium ferrocyanide, metal salts, and dyes Eriochrome Black T, Malachite Green, Methyl Orange, and Methylene Blue were purchased from Merck, India, Ltd. Deionized water was used throughout the experiment.* Aegle marmelos* was collected from Jalandhar (Punjab) India.

### 2.2. Preparation of Plant Extract

Bael leaves were collected from NIT Jalandhar campus. They were thoroughly washed thrice with deionized water and dried in oven for 48 hours at 40–50°C. Then, they were crushed into fine powder using grinder. An intense green colored extract of bael was obtained which was used in the present study.

### 2.3. Synthesis of Potassium Metal Hexacyanoferrate Nanoparticles

Here, we report the green synthesis of potassium metal hexacyanoferrate nanoparticles using* Aegle marmelos* as a biosurfactant. 100 mL of 0.1 M potassium ferrocyanide was slowly added to 100 mL of 0.1 M respective metal salts containing small amount of* Aegle marmelos* as a natural surfactant. Excess metal salt was used to get the maximum precipitation and the reaction mixture was kept undisturbed for 24 hours at ambient temperature. The precipitates were filtered and washed thoroughly with water and dried in hot air oven at 60°C. The dried product was ground and sieved with 100 mesh size sieve.

### 2.4. Characterization of KMHCF Nanoparticles

#### 2.4.1. Field-Emission Scanning Electron Microscopy (FE-SEM)

Field-Emission Scanning Electron Microscopy (FE-SEM) was performed in order to find out the surface morphology and average particle size of the synthesized KMHCF nanoparticles (Quanta 200 FEG).

#### 2.4.2. X-Ray Diffraction Analysis (XRD)

X-ray diffraction (XRD) was recorded on a PAN analytical X-PRT PRO instrument using CuK*α* (*λ* = 1.5406 Ǻ) radiation.

#### 2.4.3. Transmission Electron Microscopy (TEM)

Transmission Electron Microscopy (TEM) was conducted with Hitachi (H-7500) instrument operating at 120 kV. This study was carried out in order to find out the exact particle shape and size of the nanoparticles synthesized.

#### 2.4.4. Fourier Transform Infrared Spectroscopy Analysis

Infrared spectra of the synthesized product were recorded on Agilent instrument in the range 400–4000 cm^−1^.

#### 2.4.5. Photocatalytic Studies

Harmful organic dyes, namely, MG, EBT, MO, and MB, were degraded photochemically using KMHCF nanoparticles. The absorption spectra of the dye solutions were recorded using Agilent Pro spectrometer and the rate of degradation was observed in terms of change in intensity at *λ*_max_ of the dye. The degradation efficiency (%) has been calculated as

(1) $$\begin{array}{c} \text{Degradation efficiency }\left(\;\%\;\right)=\frac{\left(C_{0}-C\right)}{C_{0}}\times 100, \end{array}$$

where *C*_0_ is the initial concentration of dye and *C* is the concentration of dye after photoirradiation.

Different parameters such as catalyst amount, initial concentration of dye, initial pH, and temperature of the dye solution were optimized.

## 3. Results and Discussion

In 1969 formula in case of metal hexacyanoferrates was given by Dolezal and Kourim [45]. The outer sphere ion M is present in +3 oxidation state. Chandra et al. reported that the geometry of [Fe(CN)_6_]^4−^ is octahedral in which six CN^−^ ligands surround the central metal ion. CN^−^ ligands being strong field ligands will forcefully pair up with 6 electrons present in the inner sphere of Fe^2+^ and a low-spin t_2g_^6^ configuration is achieved. In addition a significant back-bonding between the metal d*π* orbitals and the antibonding p*π* orbitals of the CN^−^ ligand exists. The outer sphere metals Fe, Cu, Co, and Ni get coordinated to the nitrogen end of the cyanide ligand present inside the [Fe(CN)_6_]^4−^ sphere [46].

### 3.1. Characterization

**Figure 2:**
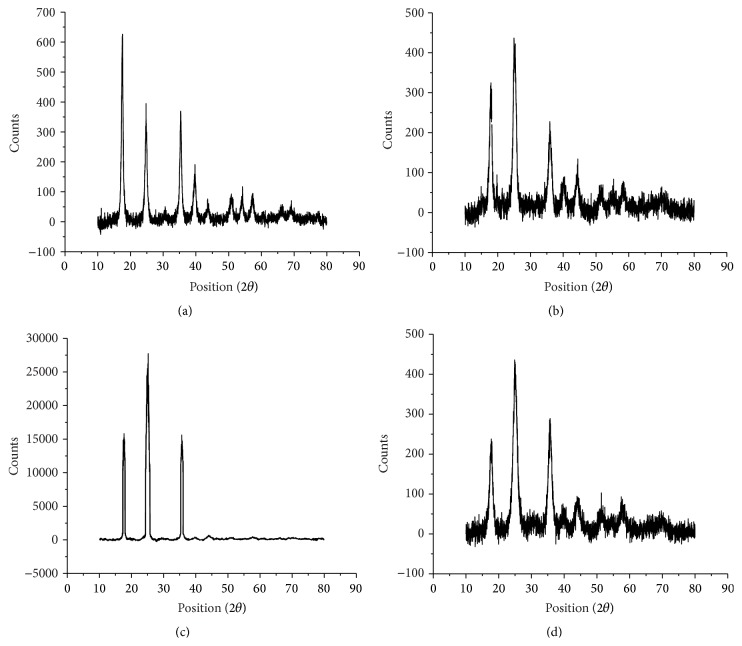
PXRD pattern of (a) FeHCF, (b) KCuHCF, (c) KCoHCF, and (d) KNiHCF nanoparticles.

Figure 2 shows the powder X-ray diffraction measurements (powder method, panalytical.X.Pert Pro) of the synthesized nanostructures. Interplanar spacing and the relative intensity data were found in good agreement with the JCPDS values (FeHCF Card number 73-0687, KCuHCF Card number 75-0024, KCoHCF Card number 75-0038, and KNiHCF Card number 23-0491). The clear and sharp X-ray peaks indicated the high crystallinity of synthesized nanomaterials.

In case of FeHCF, KCuHCF, KCoHCF, and KNiHCF the maximum relative intensity (%) was observed at 17.474°, 25.2620°, 25.044°, and 25.02° at 2*θ* scale along with 5.07114 Ǻ, 3.52264 Ǻ, 3.55276 Ǻ, and 3.557640 Ǻ  *d*-spacings, respectively. The high intensity sharp peaks clearly show the high crystallinity of synthesized KMHCF nanostructures. The 2*θ* and *d* values are given in Table 1.

**Figure 3:**
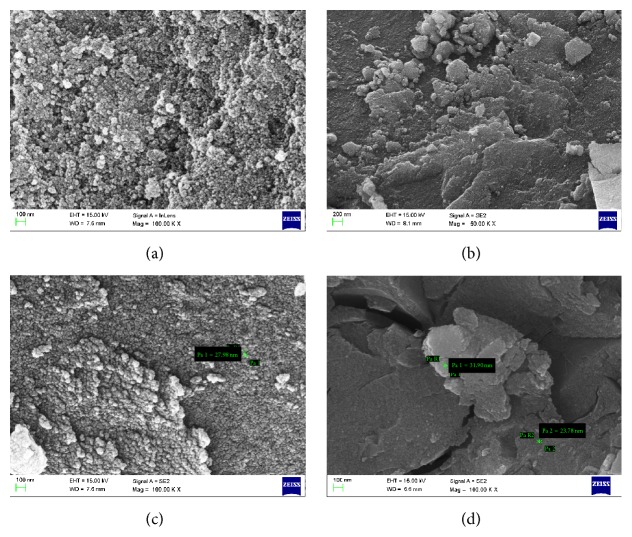
FE-SEM images of (a) Fe, (b) Ni, (c) Co, and (d) Cu HCF nanoparticles using* Aegle marmelos.*

**Figure 4:**
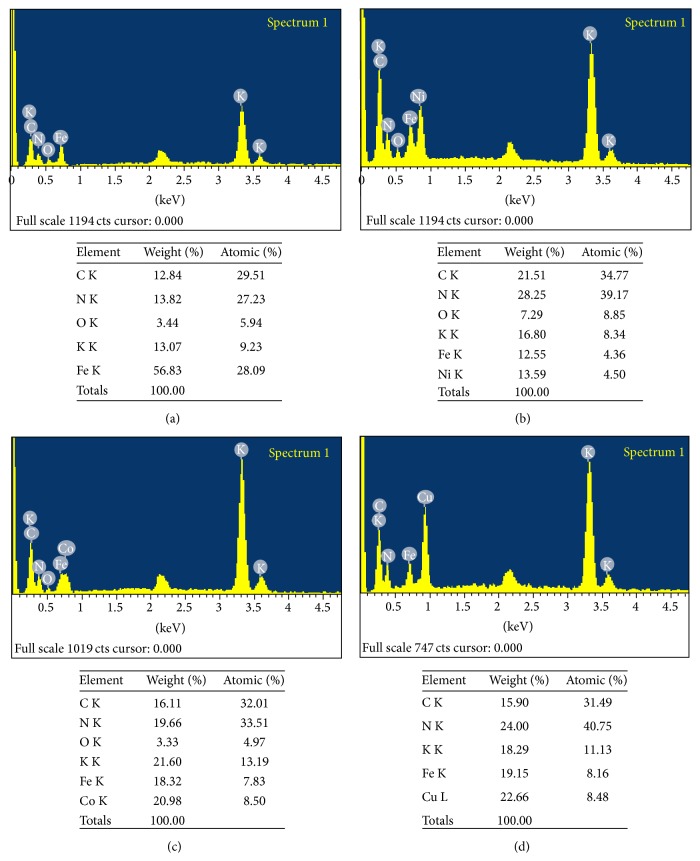
EDX pattern of (a) Fe, (b) Ni, (c) Co, and (d) Cu HCF nanoparticles using* Aegle marmelos.*

FE-SEM (Figure 3) analysis finally confirmed the synthesis of nanosized FeHCF and KMHCF nanoparticles with an average size below 50 nm. Interestingly, different transition metal nanoparticles exhibited different sizes and surface morphology. All the nanoparticles were found to be distributed uniformly throughout the ferrocyanate network. Elemental composition of the synthesized nanoparticles was confirmed through Energy Dispersive Spectroscopic (EDS) analysis (Figure 4).

In FeHCF, the C, N, O, and Fe showed weight% 12.84, 13.82, 3.44, and 56.83, whereas atomic% was found to be 29.51, 27.23, 5.94, and 28.09, respectively. In case of KCuHCF, the C, N, K, Fe, and Cu showed weight% 15.90, 24.00, 18.29, 19.15, and 22.66, along with atomic% corresponding to 31.49, 40.75, 11.13, 8.16, and 8.48, respectively. Similarly, for KCoHCF the C, N, O, K, Fe, and Co were found to contain weight% 16.11, 19.66, 3.33, 21.60, 18.32, and 20.98, whereas atomic% exhibited was 32.01, 33.51, 4.97, 13.19, 7.83, and 8.50, respectively. Also, in case of KNiHCF the C, N, O, K, Fe, and Ni were observed to have weight% 21.51, 28.25, 7.29, 16.80, 12.55, and 13.59; however atomic% was found to be 34.77, 39.17, 8.85, 8.34, 4.36, and 4.50, respectively.

**Figure 5:**
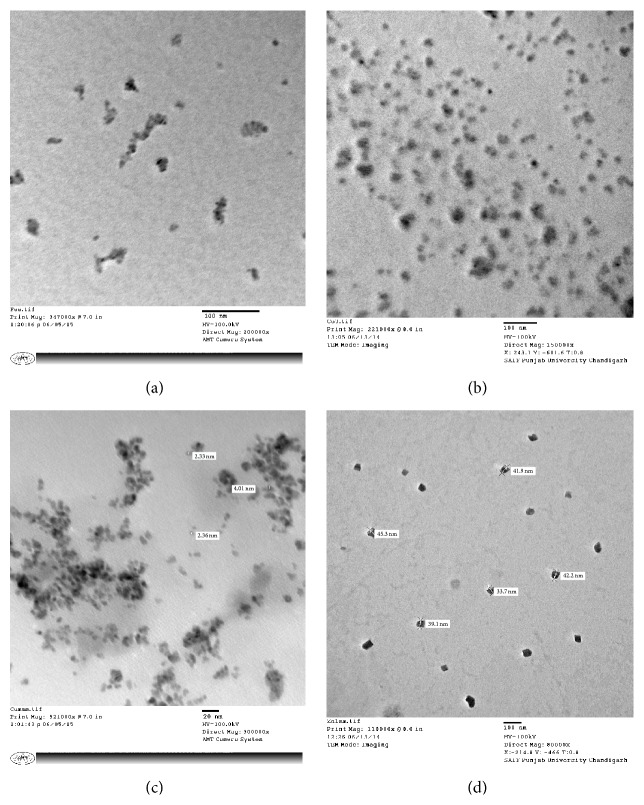
TEM images of (a) FeHCF, (b) KCoHCF, (c) KCuHCF, and (d) KNiHCF nanoparticles.

TEM was employed to characterize the exact size and shape of the PB and its analogue nanoparticles.  Figure 5 clearly indicated the presence of well dispersed nanoparticles. Depending upon the nature of transition metal ion, the KMHCF nanoparticles exhibited diverse shapes and sizes. The most interesting fact observed was that all the KMHCF nanoparticles exhibited extremely small sizes, less than 50 nm (majority of them below 10 nm). It was observed that FeHCF, KCoHCF, KCuHCF, and KNiHCF formed well dispersed and diversified shaped nanoparticles with a narrow size distribution around 6.55 nm, 21.1 nm, 4.01 nm, and 45.3 nm, respectively.

FT-IR studies were also carried out to determine the various functional groups present in the synthesized products. It is well known that the single, broad band of the *ν*_CN_ absorption of PB-structured materials in the range of 2000–2200 cm^−1^ is diagnostic of the C-bound metal ion and its oxidation state but is much less sensitive to the N-bound metal ion and its oxidation state. Peak below 400 cm^−1^ corresponds to the metal ion peak. Its frequency varies depending upon the transition metal ion.

In case of FeHCF nanoparticles, FT-IR peaks were observed at 2066 cm^−1^ (C≡N stretching), 598 cm^−1^ (Fe-C stretching), and 490 cm^−1^ (Fe-N stretching), whereas FT-IR spectra of KCoHCF nanoparticles showed peaks at 2087 cm^−1^ (C≡N stretching), 587 cm^−1^ (Fe-C stretching), and 455 cm^−1^ (Co-N stretching). In case of FT-IR spectrum of KCuHCF nanoparticles, peaks were observed at 2073 cm^−1^ (C≡N stretching), 588 cm^−1^ (Fe-C stretching), and 473 cm^−1^ (Cu-N stretching). KNiHCF nanoparticles showed stretching at 2087 cm^−1^ (C≡N stretching), 587 cm^−1^ (Fe-C stretching), and 455 cm^−1^ (Ni-N stretching). All the spectra are provided as supplementary information (Figure  1 in Supplementary Material available online at http://dx.doi.org/10.1155/2016/2715026).

### 3.2. Photocatalytic Activity

Synthesized potassium metal hexacyanoferrate nanoparticles (excluding FeHCF) were used for the degradation of MG, EBT, MO, and MB organic dyes. Absorbance measurement was carried out at regular time intervals. Different parameters such as photocatalyst dose, initial concentration of dye, initial pH, and temperature of the dye degradation were studied for getting the maximal degradation. The prime motive behind the synthesis of KMHCF nanoparticles and their evaluation as photocatalytic agents for the degradation of dyes was to achieve maximum degradation of organic dyes which are normally present in the textile effluents and are the cause of environmental hazards.

Absorbance was recorded after each interval of 20 minutes and carried out up to 2 hours. Absorbance intensity was found to decrease continuously with increase in time interval. Following trend was observed during the photocatalytic degradation of different toxic dyes: MG (96.06%, KCuHCF) > MB (94.72%, KCuHCF) > EBT (83.87%, KCuHCF) > MO (63.71%, KCuHCF). The maximum photocatalytic degradation was observed in case of MG dye using KCuHCF nanoparticles. However, minimum photodegradation was found with MO dye in presence of KCoHCF nanoparticles. Percent degradation of different dyes using various KMHCF nanoparticles is given in Table 2.

Degradation of dyes is due to the reason that nanoparticle catalysts used are semiconductor in nature which upon photoillumination generate electron-hole pairs. The generation of electron-hole pairs is responsible for the photodegradation of different dyes [47]. Further the photodegradation of dyes was found to be initial dye concentration dependent. Maximum photocatalytic degradation was observed at 5 ppm concentration in case of MG, EBT, MO, and MB. Since initially it is the adsorption of dye on the nanoparticles followed by the photocatalytic degradation process, therefore the possible explanation could be that at this dye concentration maximum number of active sites on the surface of catalyst is available for adsorption of dyes. As the concentration increases, the active sites for adsorption present on nanoparticles get blocked; thus the rate of dye degradation decreases [48]. In case of temperature dependent dye degradation studies, it was found that, with increase in temperature, relative absorption intensity decreased and negligible dye degradation was observed. This could be due to decrease in dye adsorption on the surface of nanoparticles with increase in temperature, resulting in decreased dye degradation. Thus, metal hexacyanoferrate nanoparticles in case of dye degradation act as a photocatalyst not thermocatalyst [49]. However, photodegradation of all the dyes was found to be dependent upon the concentration of the catalyst. In case of KCuHCF nanoparticles at the concentration of 15 mg per 10 mL of each dye maximum photodegradation of 96.06%, 83.87%, 94.72%, and 63.71% was found in case of MG, EBT, MB, and MO, respectively. However, in case of KNiHCF nanoparticles, maximum photodegradation of MG, EBT, MO, and MB was found to be 95%, 79.84%, 59.42%, and 91.35%, respectively. KCoHCF nanoparticles degraded the dyes MG, EBT, MO, and MB to the extent of 90.87%, 75.81%, 58.20%, and 89.28%, respectively. This could be due to the reason that, at preoptimized dye concentration, too high or low dose of the catalyst was not a favorable condition. A moderate catalyst concentration (15 mg/10 mL) was found to be optimal for the decomposition of MG, EBT, MO, and MB [50]. pH was found to play an important role in the photocatalytic degradation of dyes. Dye degradation was found to increase with increase in pH. However, maximum degradation was found at pH 7.0. Further increase in pH resulted in decreased dye degradation. This could be due to the ion screening effects of H^+^ ions under acidic conditions and OH^−^ ions in alkaline medium. Ion screening effects of H^+^ ions at low pH and OH^−^ ions under alkaline conditions have the adverse impact on the adsorption of dyes on the KMHCF nanoparticles and hence decreased dye degradation was observed [47]. KMHCF nanoparticles are insoluble in water as well as fairly stable in acids like HCl, HNO_3_, and H_2_SO_4_ as well as bases like NaOH and KOH. Thus, after the completion of dye degradation reactions, catalysts were recovered and could be used again for similar further reactions.

### 3.3. Adsorption Isotherms

**Figure 6:**
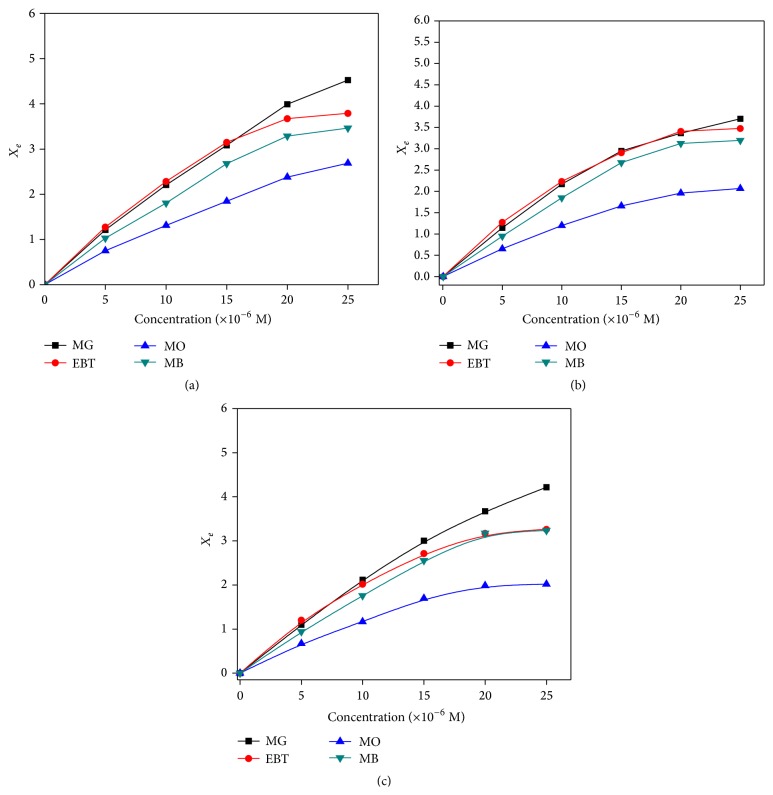
Adsorption isotherms for adsorption of MG, EBT, MO, and MB dyes on (a) KCuHCF, (b) KNiHCF, and (c) KCoHCF nanoparticles.

**Figure 7:**
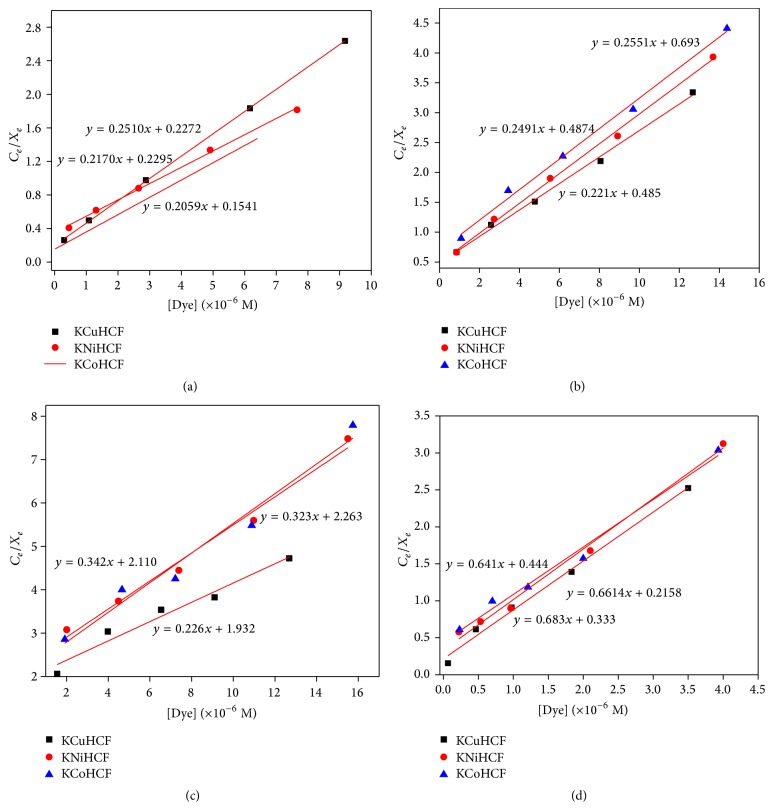
Langmuir isotherms for adsorption of (a) MG, (b) EBT, (c) MO, and (d) MB on KMHCF nanoparticles.

Adsorption isotherms (Figure 6) of MG, EBT, MO, and MB in the present case clearly show that adsorption is fast in all the cases and the isotherms are regular, positive, and concave to the concentration axis. Adsorption data can be represented through Langmuir adsorption isotherms which assume the formation of a monolayer of solute molecules on the surface of the adsorbent [51]. A typical graph of *C*_*e*_/*X*_*e*_ v/s *C*_*e*_ of all the dyes is a straight line (Figure 7). The adsorption data was fitted in Langmuir adsorption equation:

(2) $$\begin{array}{c} \frac{C_{e}}{X_{e}}=\frac{1}{k_{L}X_{m}}+\frac{C_{e}}{X_{m}} \end{array}$$

or

(3) $$\begin{array}{c} \frac{1}{X_{e}}=\frac{1}{C_{e}}\left(\;\frac{1}{k_{L}X_{m}}\;\right)+\frac{1}{X_{m}}, \end{array}$$

where *C*_*e*_ is the equilibrium concentration of the dye solution; *X*_*e*_ is the amount of dye adsorbed per gram weight of adsorbent; *X*_*m*_ is the amount of dye adsorbed at saturation; *k*_*L*_ is the Langmuir adsorption constant. *k*_*L*_ and *X*_*m*_ values were determined and shown in Table 3. In case of dye adsorption on KMHCF nanoparticles, *X*_*m*_ values followed the trend: 4.856 mg/g (Malchite Green adsorbed on KCuHCF nanoparticles, i.e., MG-ad-KCuHCF) > 4.608 mg/g (MG-ad-KCoHCF) > 4.525 mg/g (EBT-ad-KCuHCF) > 4.49 mg/g (MO-ad-KCuHCF) > 4.014 mg/g (EBT-ad-KNiHCF) > 3.984 mg/g (MG-ad-KNiHCF) > 3.920 mg/g (EBT-ad-KCoHCF) > 3.895 mg/g (MB-ad-KCoHCF) > 3.799 mg/g (MB-ad-KCuHCF) > 3.658 mg/g (MB-ad-KNiHCF) > 3.094 mg/g (MO-ad-KNiHCF) > 2.923 mg/g (MO-ad-KCoHCF). However, *k*_*L*_ values were found to follow the trend 8.54 dm^3^/mol (MO-ad-KCuHCF) > 7.00 dm^3^/mol (MO-ad-KNiHCF) > 6.170 dm^3^/mol (MO-ad-KCoHCF) > 2.716 dm^3^/mol (EBT-ad-KCoHCF) > 2.194 dm^3^/mol (EBT-ad-KCuHCF) > 1.956 dm^3^/mol (EBT-ad-KNiHCF) > 1.057 dm^3^/mol (MG-ad-KCoHCF) > 0.905 dm^3^/mol (MG-ad-KNiHCF) > 0.7483 dm^3^/mol (MG-ad-KCuHCF) > 0.692 dm^3^/mol (MB-ad-KCoHCF) > 0.487 dm^3^/mol (MB-ad-KNiHCF) > 0.342 dm^3^/mol (MB-ad-KCuHCF).

Maximum *k*_*L*_ value was found in case of adsorption of MO on KCuHCF nanoparticles whereas minimum value was observed with the adsorption of MB on KCuHCF nanostructures. Further it has been observed that maximum *k*_*L*_ values were shown by the adsorption of MO dye on different KMHCF nanoparticles, whereas minimum *k*_*L*_ values were found with the adsorption of MB on different KMHCF nanoparticles. Higher *X*_*m*_ values, that is, adsorption of MG on KCuHCF nanoparticles, may be due to the interaction of N-atoms of MG with the nanoparticles [52].

**Figure 8:**
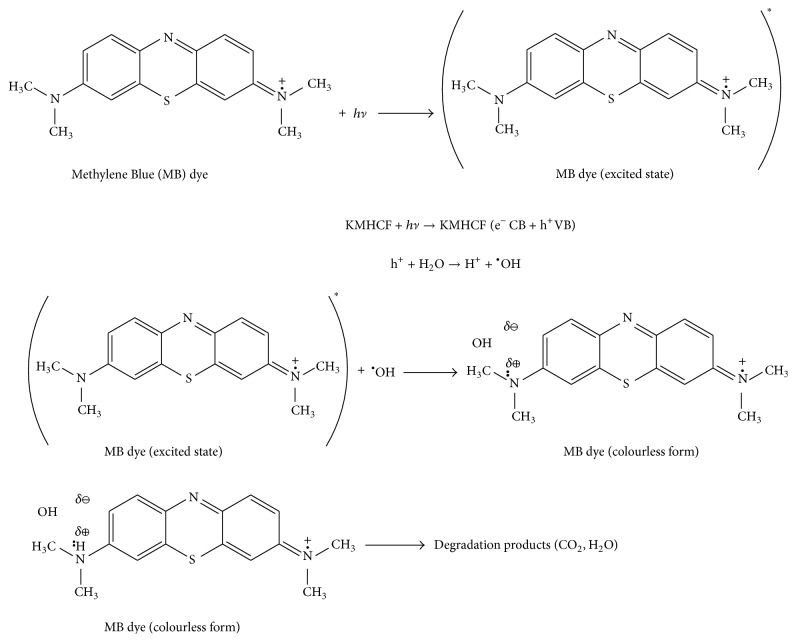
Photocatalytic degradation of MB in presence of KMHCF nanoparticles.

Since KMHCFs are semiconducting materials and molecular excitation takes place easily, thereby generating electrons and holes in between conduction and valence bands, this is followed by a series of chemical reactions leading to the formation of hydroxyl free radicals. It is the OH^•^ which leads to the photodegradation of harmful organic dyes. Since it is the adsorption of the dye which takes place initially on the surface of adsorbent and afterwards is followed by dye degradation process, therefore, the proposed reaction sequence for the degradation of toxic organic dye MB in presence of KMHCF nanoparticles is given in Figure 8 (degradation mechanism for MG, EBT, and MO has been given as supplementary information as Figures  2,  3, and 4).

### 3.4. Thermodynamic Parameters

Dependence of *k*_*L*_ (equilibrium constant) with temperature can be used to predict thermodynamics parameters, namely, Gibb's free energy change (Δ*G*°), enthalpy change (Δ*H*°), and entropy change (Δ*S*°) associated with the dye adsorption process. These parameters were determined using the following equations:

(4) ΔG°=−RTkL,ln⁡kL=−ΔG°RT=−ΔH°RT+ΔS°R.

Plot of ln⁡*k*_*L*_ v/s 1/*T* yields a straight line. The slope as well as intercept of this plot gives the values of Δ*H*° and Δ*S*°, respectively. The energy parameters Δ*G*°, Δ*H*°, and Δ*S*° for MG, EBT, MO, and MB are given in Table 4.

The negative values of Δ*G*° at all temperatures suggest that the dye adsorption on the nanoparticles is spontaneous. The negative values of Δ*H*° indicate that the nature of adsorption is exothermic. The positive values of Δ*S*° suggest increased randomness at the solid/liquid interface during the adsorption of dyes onto different KMHCF nanostructures.

## 4. Conclusions

Monodisperse KMHCF nanoparticles (FeHCF, KCuHCF, KNiHCF, and KCoHCF) using* Aegle marmelos* (bael) as a biosurfactant were synthesized through a green route. Plant based biosurfactant provides an environmentally friendly method since the route eliminates the use of toxic chemicals. This bioassisted approach led to instantaneous formation of nanoparticles with uniform distribution at room temperature. Synthesized nanoparticles were evaluated as photocatalyst for the degradation of MG, EBT, MO, and MB dyes. Different process parameters like photocatalyst dose, initial dye concentration, pH, and temperature were studied in order to get the maximum dye degradation. MG was found to degrade to the maximum extent (96.06%) whereas minimum degradation was found with MO (58.20%) in presence of KCuHCF, KCoHCF nanoparticles, respectively. EBT, MB, and MO were found to show maximum degradation of 83.87%, 94.72%, and 63.71% using KCuHCF nanoparticles; adsorbate dose 15 mg; each dye concentration 5 ppm and temperature, ambient. Thus, KMHCF nanoparticles are promising agents for the treatment of textile effluents containing harmful organic dyes.

## Supplementary Material

Figure 1 in Supplementary information describes the FT-IR spectra of all the synthesized KMHCF nanoparticles. A broad band in the range of 2000–2200 cm^−1^ is diagnostic of the *ν*_CN_ absorption of PB and its analogues. Peak below 400 cm^−1^ corresponds to the metal ion peak, whose frequency varies depending upon the transition metal ion. Also, degradation mechanisms of toxic organic dyes MG, EBT and MO in the presence of KMHCF nanoparticles are given as Figures 2, 3, and 4. Since KMHCF nanoparticles are semiconducting in nature, hence, molecular excitation takes place easily. Thus, electrons and holes are generated in conduction and valence bands, respectively. A series of chemical reactions occur which ultimately result in the formation of hydroxyl free radicals. This OH^•^ leads to the photodegradation of harmful organic dyes.

## Figures and Tables

**Table 1:** PXRD parameters of FeHCF and other different KMHCF nanostructures.

Sample	Position (2*θ*)	Height (cts)	FWHM	*d*-spacing (Ǻ)	Relative intensity (%)
FeHCF	17.474	425 (7)	0.525 (9)	5.07114	100.00
	24.740	227 (8)	0.74 (1)	3.59570	53.48
	35.309	237 (5)	0.61 (1)	2.53995	55.93
	39.592	99 (4)	0.89 (2)	2.27449	23.33

KCuHCF	17.8270	198.38	0.8868	4.97150	90.79
	25.2620	218.50	0.9642	3.52264	100.00
	35.9171	124.35	1.1609	2.49830	56.91

KCoHCF	17.685	102 (3)	0.82 (2)	5.01098	58.98
	25.044	173 (3)	1.04 (2)	3.55276	100.00
	35.69	96 (4)	0.97 (3)	2.51360	55.61

KNiHCF	17.46	93 (14)	0.90 (9)	5.07388	39.51
	25.02	234 (22)	1.61 (5)	3.55640	100.00
	35.657	171 (4)	1.30 (2)	2.51592	73.06

**Table 2:** Degradation of different dyes using different KMHCF nanoparticles.

Dye	Nanostructures	% dye degradation
MG	KCuHCF	96.06
	KNiHCF	95.00
	KCoHCF	90.87

EBT	KCuHCF	83.87
	KNiHCF	79.84
	KCoHCF	75.81

MO	KCuHCF	63.67
	KNiHCF	59.42
	KCoHCF	58.20

MB	KCuHCF	94.72
	KNiHCF	91.35
	KCoHCF	89.28

MG = Malachite Green, EBT = Eriochrome Black T, MO = Methyl Orange, and MB = Methylene Blue.

**Table 3:** Langmuir constants for different dyes adsorption on various KMHCF nanoparticles.

Dye	Sample	*X*_*m*_ (mg/g)	*k*_*L*_ (dm^3^/mol)
MG	KCuHCF	4.856	0.7483
	KNiHCF	3.984	0.9051
	KCoHCF	4.608	1.057

EBT	KCuHCF	4.524	2.194
	KNiHCF	4.014	1.956
	KCoHCF	3.920	2.716

MO	KCuHCF	4.49	8.54
	KNiHCF	3.094	7.00
	KCoHCF	2.923	6.170

MB	KCuHCF	3.799	0.342
	KNiHCF	3.658	0.487
	KCoHCF	3.895	0.692

MG = Malachite Green, EBT = Eriochrome Black T, MO = Methyl Orange, and MB = Methylene Blue; *X*_*m*_ = amount of dye adsorbed; *k*_*L*_ = Langmuir adsorption constant.

**Table 4:** Thermodynamic parameters of different dyes adsorption on various KMHCF nanoparticles.

Dye	Sample	Thermodynamic parameters				
		−Δ*G*°(kJ mol^−1^)			Δ*H*° (kJ mol^−1^)	Δ*S*° (kJ mol^−1^ K^−1^)
		30°C	40°C	50°C		
MG	KCuHCF	9.387	10.271	10.768	−1361.42	8.237
	KNiHCF	9.866	10.749	11.440	−1651.25	9.376
	KCoHCF	10.257	10.868	11.563	−1112.78	7.738

EBT	KCuHCF	12.097	12.604	13.051	−277.82	5.722
	KNiHCF	11.807	12.398	13.023	−773.59	7.237
	KCoHCF	12.634	13.164	13.773	−537.55	6.784

MO	KCuHCF	15.521	16.093	16.659	−202.054	6.828
	KNiHCF	15.019	15.555	16.102	−161.480	6.494
	KCoHCF	14.702	15.233	15.752	−142.235	6.306

MB	KCuHCF	7.414	7.814	8.250	−612.145	4.961
	KNiHCF	8.305	8.804	9.189	−600.317	5.284
	KCoHCF	9.190	9.643	10.027	−411.435	5.009

Δ*G*°= Gibb's free energy change, Δ*H*° = enthalpy change, and Δ*S*° = entropy change.
