# Supplementary material for: Aegle marmelos Mediated Green Synthesis of Different Nanostructured Metal Hexacyanoferrates: Activity against Photodegradation of Harmful Organic Dyes
Source: Scientifica (Cairo). 2016 Feb 29;2016:2715026. doi: 10.1155/2016/2715026 (PMC4789527; doi:10.1155/2016/2715026)
Supplement: Supplementary file 1 — Figure 1 in Supplementary information describes the FT-IR spectra of all the synthesized KMHCF nanoparticles. A broad band in the range of 2000–2200 cm−1 is diagnostic of the ν CN absorption of PB and its analogues. Peak below 400 cm−1 corresponds to the metal ion peak, whose frequency varies depending upon the transition metal ion. Also, degradation mechanisms of toxic organic dyes MG, EBT and MO in the presence of KMHCF nanoparticles are given as Figures 2, 3, and 4. Since KMHCF nanoparticles are semiconducting in nature, hence, molecular excitation takes place easily. Thus, electrons and holes are generated in conduction and valence bands, respectively. A series of chemical reactions occur which ultimately result in the formation of hydroxyl free radicals. This OH• leads to the photodegradation of harmful organic dyes. [file 2715026.f1.docx]

**Supplementary Information**

***Aegle marmelos* mediated green synthesis of different nano-structured metal hexacyanoferrates: Activity against photodegradation of harmful organic dyes**

**Vidhisha Jassal, Uma Shanker* and B S Kaith**

**^*^Department of Chemistry**

**Dr B R Ambedkar National Institute of Technology**

**Jalandhar, Punjab, India-144011**

*** Corresponding Author**

**Dr Uma Shanker**

**(Assistant Professor)**

**Office Number-CE-306**

**Department of Chemistry**

**Dr B R Ambedkar National Institute of Technology Jalandhar,**

**Jalandhar, Punjab, India-144011**

**Email:** [**shankeru@nitj.ac.in**](mailto:shankeru@nitj.ac.in)**,** [**umaorganic29@gmail.com**](mailto:umaorganic29@gmail.com)

**Contact number: +91- 7837-588-168 (Mobile)**

**+91-0181-269-301-2258 (Office)**

**Fax: +91-0181-269-0932**

1. (b)

(c) (d)

**Figure 1. FT-IR spectra of (a) FeHCF (b) KCoHCF (c) KCuHCF (d) KNiHCF nanoparticles**

**Figure 2. Photocatalytic degradation of MG in presence of KMHCF nanoparticles**

**Figure 3. Photocatalytic degradation of EBT in presence of KMHCF nanoparticles**

**Figure 4. Photocatalytic degradation of MO in presence of KMHCF nanoparticles**
